# Predictive Biomarkers of Severe Immune-Related Adverse Events With Immune Checkpoint Inhibitors: Prevention, Underlying Causes, Intensity, and Consequences

**DOI:** 10.3389/fmed.2022.908752

**Published:** 2022-06-14

**Authors:** Ana Cardeña-Gutiérrez, Mónica López Barahona

**Affiliations:** ^1^MedicalOncologyDepartment, Hospital Universitario Nuestra Señora de Candelaria, Santa Cruz de Tenerife, Spain; ^2^Centro Estudios Biosanitarios, Madrid, Spain

**Keywords:** immune checkpoint proteins, immune-related adverse event (irAE), biomarker, autoimmunity, severe toxicity

## Abstract

Immune checkpoint inhibitors (ICIs) have dramatically transformed oncology by prolonging overall survival and yielding better patient tolerance compared to other chemotherapeutic agents. However, numerous questions remain unanswered about the toxicity profile of ICIs, its relationship with the treatment response, and causes underlying the excellent treatment response in some patients, while recalcitrance in others. Research groups have continued to seek biomarkers that may permit the identification of treatment responders and predict toxicity to facilitate cessation of immunotherapy before the development of severe toxicity. However, some studies have found associations between serious adverse events and longer survivorship. The research question entailed determining whether a biomarker is needed to predict severe immune-related adverse events prior to their development or whether providing early treatment for toxicity would inhibit the immune system from attaining a long-lasting anti-tumor effect. Therefore, this review conducted an in-depth analysis into the molecular basis of these observations.

## Introduction

Immune checkpoint inhibitors (ICIs) have sparked a massive revolution in oncology. Immune checkpoints are a group of membrane receptors present on cytotoxic T lymphocytes whose function is to prevent an indefinite immune response that could severely damage healthy host tissue. Programmed cell death protein 1 (PD1) and cytotoxic T-lymphocyte antigen 4 (CTLA-4) are the most studied immune checkpoints. ICIs are monoclonal antibodies that target PD-1/programmed death ligand 1 (PD-L1) or CTLA-4, reactivating the anti-tumor immune response that is inhibited by the overexpression of these proteins by tumor cells (1). However, not all patients respond to immune checkpoint blockade. Response rates range from 13 to 40%, depending on monotherapy or combination treatment and the primary tumor (1). Hence, it imperative to discover biomarkers that can aid in predicting the treatment response to avoid the administration of ineffective drugs, which are also exorbitantly expensive. Despite tremendous efforts in this field, PD-L1 expression, tumor mutational burden (TMB), and microsatellite instability are the only predictors available for use in routine clinical practice (2), although their specificity is not ideal.

Moreover, even though the toxicity profile of ICIs is better than that of chemotherapy, the rate of adverse effects is significantly high with ICIs. Severe treatment-related toxicity was observed in 55% of patients treated with the combination of PD-L1 and CTLA4 inhibitors (3) (grades 3–4 according to the Common Terminology Criteria for Adverse Events version 5) (4). Colitis, rash, and hypophysitis are the most frequent adverse effects of CTLA4 inhibitors, whereas arthralgia, pneumonitis, vitiligo, and hypothyroidism are most frequent with PD-L1 inhibitors (5), with high temporal unpredictability (6). However, some studies have shown that immune-related adverse events (irAEs) could be predictors of the anti-tumor response (7, 8).

The individual irAEs evoked by ICIs bear striking similarities to classic autoimmune diseases (9),with the main bulk of evidence being focused on immune-related colitis (10, 11). The mechanisms that trigger irAE development are incompletely understood, but are chiefly related to the loss of peripheral tolerance and increase in self-reactive T-cell clones (12). They are often poorly reported in clinical trials, and until recently, principal knowledge about their development was derived from the retrospective studies, whose *a posteriori* nature precludes the collection of samples and analysis of possible triggers. Usually, irAEs develop in 1 organ at a time and are considered to be dose independent; however, with anti-CTLA4, recent studies show differences depending on the administered dose of ipilimumab (13). Moreover, irAEs can appear even months after cessation of the drug, which may be challenging to identify and treat in routine clinical practice (6, 12).

The initial treatment for severe irAEs entails the administration of high-dose corticosteroids (specifically, methylprednisolone 1 mg/kg/day) (3, 14). Other immunomodulators, such as infliximab, vedolizumab, tocilizumab, mycophenolate, etc., can be added, if the irAE cannot be controlled with corticosteroids alone (Table 1). Usually, treatment must be stopped if the patient develops severe toxicity, but it is not always linked to cessation of the anti-tumor benefit, and may even have the opposite effect, i.e., years of recurrence-free survival without any treatment (15). However, other patients experience an explosion of disease after the administration of high-dose corticosteroids or other immunosuppressants (16). Therefore, this treatment was initially contraindicated in patients treated with ICIs, because an abrupt loss of effectivity was anticipated (17).

**Table 1:** Management of the most frequent severe irAEs (14) and biomarkers that may predict them.

**irAEs, all grades** **(% PD-L1/ CTLA-4/ combination)** **Median time to onset (41)**	**Common management (grade ≥3) (14)**	**Special management considerations (14)**	**Biomarker: immune cells**		**Biomarker: cytokines** **(↑except indicated)**	**Other potential biomarkers**
**Colitis** **(<19, 13–54, 29)** **38 days**	Consider patient admission	Infliximab or vedolizumab	↑CD4TH17 ↓Tregs (41, 42) ↑CD177 and CEACAM1 genes (46)		IL-17 (31, 42) ↓ IL-6 (31) IL-8 (31, 42)	Microbiome (21, 79) ↑Faecalibacterium, ↑Firmicutes, ↓Bacteroidetes (colitis) TGFβ signature (58) NLR (better accuracy for pneumonitis) (80) Eosinophils (41) Lymphocytes >2000 (41) Sarcopenia (58) Body mass index (60) Vitamin D (on investigation)
**Dermatitis** Incidence of all dermatological irAEs: (17–37, 37–70, 48) 25 days	Steroids 1–2 mg/kg/day until grade 1, followed by a tapered dose for 4–6 weeks*	Topical emollients, corticosteroids, oral antihistamines Consider phototherapy	-	↓Circulating B cells ↑CD21^lo^ B cells/plasmablasts (44)	IL-6 IL-10 (19)	
**Arthritis** (6–12, 5, 11) 3 months	Consider indefinite suspension of the drug *	Long- term administration of TNF inhibitor or consider tocilizumab (81)	↓CD8 effectors (12)		IL-6	
**Pneumonitis** (<1, 2,7, 10) 3 months		Infliximab or mycophenolate mofetil IV/IVIG or cyclophosphamide	↑CD4 TH2 (12)		**-**	
**Thyroid disorders** Hypothyroidism (6, 4, 13), Hyperthyroidism (3, 2, 8) 14–73 days		Hold the drug until symptoms resolve to baseline with appropriate therapy Consider IV levothyroxine for myxoedema, steroids and supportive care	↑CD4 TH17 (12)		-	

^*^Except thyroid disorders.

*(% PD-L1, CTLA-4, or a combination of the 2): Percentage of incidence of these irAEs according to the administered drug(s) (41)*.

*irAEs, immune-related adverse events; PD-L1, programmed cell death ligand 1; CTLA-4, cytotoxic T-lymphocyte antigen 4; NLR, neutrophil to lymphocyte ratio*.

The principal hypothesis that motivated this research is whether a biomarker should be sought to predict severe irAEs prior to their development, or if early treatment for toxicity will inhibit the immune system from attaining a long-lasting anti-tumor effect. This study delved into the molecular basis of these observations, reviewed the pathogenesis of irAEs, and sought biomarkers that could specifically predict severe toxicity. Furthermore, it attempted to elucidate the molecular link between toxicity and the anti-tumor response, and discussed the need for these biomarkers in clinical settings and the implications of possible preventive treatment for irAEs.

## Immune-Related Adverse Events: Molecular Basis

Immune checkpoints play a fundamental role in maintaining immunologic homeostasis (6). Therefore, the blockade of these checkpoints may increase the anti-tumor activity of the immune system, which is accompanied by the risk of the loss of self-tolerance, leading to the occurrence of irAEs, causing damage to normal cells and tissues.CTLA4 modulates the immune response in theearly stages, while PD-1 acts later in the immunologic cycle (1, 12). CTLA-4 blockade induces expansion of the inducible T-cell costimulatory Th1-like CD4 effect or as well as exhausted-like TCD8+ cells, while PD-1 blockade primarily induces expansion of exhausted-like tumor infiltrating TCD8+ cells (18).

The deficiency of CTLA-4 leads to severe autoimmune diseases (colitis and myocarditis) characterized by T-cell infiltration in murine models. This phenomenon also occurs with the loss of PD-1, but is less straight forward with genetic strain differences, and may be accompanied by the development of late-onset autoimmune diseases (such as lupus-like disease) (19).

The self-tolerance of the immune system, in which regulatory T (Treg) cells play a fundamental role, can be lost in several ways. Tregs are a subgroup of CD4 + T lymphocytes that maintain immune tolerance. Usually, a higher count of Tregs in peripheral blood is related with poor prognosis for several cancers (20). Nuclear factor kappa B (NF-κB) activation is essential for Treg-induced homeostasis, and Treg and effector T-cell expansion (21). Constitutive activation of NF-κB-induced kinase (NIK) on Tregs induces alteration of its functions and genetic signature (GITR+CD25+Foxp3+), leading to development of autoimmune diseases (20). CD25+ T and CD25– lymphocytes inhibit the development of autoimmunity, which could also be evoked by FOXP3 expression, which, in turn, increases Treg and M2 macrophage infiltration (immunosuppression), tipping the balance in favor of the tumor cells. Polymorphisms in the Foxp3 locus affect Foxp3 expression and can influence Treg cell function (22). The increase in NOTCH3 also plays a role in decreasing the TMB, the GEP-gene expression profile scores, and the TCD8+ activated lymphocytic infiltration. This mechanism is correlated with adenosine 2A receptor (ADORA2A) and CD276 (B7-H3) expression (23), both of which possess potential therapeutic effects (24, 25). Adenosine, which is generated in the tumor microenvironment (TME), inhibits the anti-tumor function of various immune cells, such as cytotoxic T cells and natural killer (NK) cells. Moreover, ADORA2A is implicated in the upregulation of inhibitory cytokines, such as transforming growth factor-beta (TGF-β) and inhibitory receptors, such as PD-1 itself. Interactions with FOXP3 stimulate the transformation of CD4+ T-cells into Treg cells, thus inhibiting the immune response (26).

Furthermore, T-cell activation is markedly sensitive to the depletion of glutamine and glucose, and the exogenous uptake of serine and alanine (27). Effect or T cells are consequently sensitive to the oxidative stress in the TME, which can induce the exhausted phenotype (27), which may be implicated in response and toxicity.

However, T cells are not the only protagonists involved in the development of irAEs. The possible role of cytokines and other immune cells involved in the maintenance of self-tolerance, and consequently, irAE development, such as the previously mentioned NK cells, B cells, and autoantibodies, which are products of the humoral immune system, should not be forgotten.

First, cytokines such as the IL-12 family (IL-12, IL-23, IL-27, and IL-35) may be related to both tumor immunity and autoimmunity, necessitating examination of their modulation in irAEs (28). The other cytokines related to immune inhibition include IL-10, IL-4, IL-6, and IL-13 (1), and TGF-β, which is correlated with FoxP3 expression and T-reg infiltration and immunesuppression in some models (29).

Second, given that autoantibodies are associated with the development of some autoimmune diseases, such as Hashimoto's thyroiditis and rheumatoid arthritis, autoantibodies can be considered as a potential cause of irAEs (30). However, not all antibodies play a role in the pathogenesis of irAEs.

Therefore, the development of irAEs could be related to the following mechanisms: surge in T cell activity against antigens that are present in tumors and healthy tissue, elevation in the levels of pre-existing antibodies or inflammatory cytokines, or enhancement of complement-mediated inflammation due to direct binding of anti-CTLA4 antibodies with CTLA4 expressed on normal tissue (6). These interactions could cause cellular toxicity via antibody-dependent cellular cytotoxicity, antibody-dependent cellular phagocytosis, and complement-dependent cytotoxicity (9).

The molecular basis of irAEs differs depending on the individual drug (31). Usually, anti PD-1toxicity is mediated by auto antibodies that already exist in patients and are stimulated after the initiation of ICIs (32). Therefore, irAEs, such as thyroid disorders or vitiligo, are more frequent with anti-PD-1/PD-L1 drugs. Hence, we may infer that patients with a history of spontaneous autoimmune diseases would experience irAEs with greater frequency and (possibly) greater severity, which may or may not be related to the underlying autoimmune disease. The findings of studies and case reports in this regard were controversial (33–36). The reported frequency of disease flares was higher with anti-PD-1/PD-L1 drugs (62 vs. 36%), while that of *de novo* irAEs was higher with ipilimumab (42 vs. 26%), which could be related to the previous observation on autoantibodies (37). However, although irAEs and flares are frequent among patients with autoimmune diseases (especially those with rheumatoid arthritis), their toxicities are usually manageable even without cessation of ICI therapy (38). Further evidence and guidelines are required in the future to fully understand the mechanisms underlying irAEs and autoimmunity, and advise clinicians on the safe prescription of ICIs in this context.

We must reiterate that some irAEs appear more frequently when ICIs are used for the treatment of specific tumors, albeit not in all patients. For example, the incidence of vitiligo is higher in patients with melanoma (39). Since ICIs increase the anti-tumor response via melanocytes, it is not surprising that the occurrence of vitiligo may be associated with an increased anti-tumor response (6, 40).

Colitis is another example highlighting how the development of different irAEs depends on the culprit drug. If the irAE is induced by anti-PD-L1, CD8+ T lymphocyte infiltration is observed in the intestinal mucosa, whereas irAEs caused by anti-CTLA4 are characterized by the predominance of CD4+T cells and elevation in TNF-α levels (11). Lower levels of TNF-α in the intestinal mucosa are related to better sensitivity to corticosteroids (11).

## Biomarkers for Severe Toxicity

We sought biomarkers to predict irAE occurrence before their induction, in order to facilitate early treatment to avoid severe (grade 3) and life-threating (grade 4) toxicity. The more promising ones are mentioned in this section, although none of these have been validated yet, and larger prospective studies focusing on this aspect are vital.

The first potential biomarker is related to enhanced T-cell activity against antigens present in tumor and healthy tissue; specific TCR sequences predispose cancer patients to organ-specific toxicities. For example, a lower proportion of CD8+ effector cells is associated with arthritis, while a higher proportion of CD4 TH2 cells and CD4 TH17 cells at baseline is related to pneumonitis and thyroiditis, respectively (12). It is logical to infer that a reduction in the proportion of Tregs could be related to higher toxicity, but limited data is available on its predictive ability for colitis (41, 42). Thus, the future direction for tumor immunotherapy lies in enhancing the function of tumor-specific T cells rather than that of other T-cell subtypes (43).

Furthermore, circulating B cells may be useful for predicting irAEs. Patients with melanoma treated with ICIs who experienced a 30% or greater reduction in the baseline levels of total circulating B cells, and increase in CD21^lo^ B cells or plasma blasts, were significantly more likely to develop high-grade IRAEs than those without B cell changes (44). Interestingly, PD1 expression was higher in CD21^lo^ B cells (45). Further studies are needed to validate these observations.

Additionally, the infiltration of digestive neutrophils into the colon during treatment is associated with digestive toxicity with anti-CTLA-4, in addition to the increased expression of the CD177 and CEACAM1 genes, which are markers of neutrophil activation (46).

First, the following useful biomarkers should be mentioned, which are simple and inexpensive to detect the neutrophil to lymphocyte ratio, which is elevated in patients who develop grade 3 and 4 pneumonitis and colitis after anti-PD-1;the absolute eosinophil count, which increases before the onset of >grade 2 endocrine disorders; and the absolute lymphocyte count (>2,000/mL). These parameters are related to irAEs, albeit without any specificity (41, 46), and can be easily altered with the incidence of other conditions such as infectious diseases, which may alter prognostication.

Second, humoral biomarkers should also be considered, since elevated levels of pre-existing antibodies or inflammatory cytokines act as triggers for the development of irAEs; IL-6, IL-17, and sCD163 are significantly associated with irAEs in cancer patients treated with ICIs (7, 47). CD4 TH-17 cells secreting IL-17, IL-6, and IL-8 appear in patients who develop grade >3 colitis (with anti CTLA-4) (42, 46). The elevated levels of IL-6 and IL-10 are also linked with dermatological irAEs (19), while lower levels of IL-6 are reportedly associated with colitis (31).

Although the higher levels of autoantibodies have been linked to the irAE development, the relationship between auto antibodies and the pathogenesis of toxicities is unclear. Enhanced T-cell activation may be the most plausible trigger for irAEs, while the humoral immune system may play a supporting role. These phenomena can be measured using protein microarrays, akin to those for autoimmune diseases (48). However, anti-thyroid peroxidase (49) is the only antibody that can be employed in routine in daily clinical practice to predict irAEs. Furthermore, recent studies have found no association between baseline auto antibodies and irAE severity (30). These findings have precluded their use for the prediction of severe toxicity, and consequently, prophylactic treatment.

Finally, a few studies have posited gene signatures as a potential predictive measure for irAE incidence and severity, at least for immune-related colitis (50). A strategy that combined pharmacovigilance data with omics data identified 2 additional potential biomarkers associated with the use of PD-1/PD-L1 agents, viz. lymphocyte cytosolic protein 1, which is involved in T-cell activation, and adenosine diphosphate dependent glucokinase, which mediates the metabolic shift during T-cell activation (51). Nevertheless, these findings were derived from a small sample, and further investigations are needed to validate these biomarkers.

The microbiome, as well as body mass index (BMI) and body composition, are the two intriguing potential biomarkers under investigation.

Fecal microbiome transplantation (FMT) has emerged as a treatment for immune-related colitis. A study showed reconstitution of the gut microbiota and elevation in Treg cells within the colonic mucosa with FMT (52). The baseline gut microbiota enriched with Faecalibacterium and other Firmicutes were found to be associated with the clinical response and CTLA-4-induced enterocolitis (42). The 2 studies reported that a low abundance of Bacteroidetes was associated with colitis. Nevertheless, to date, the studies that analyzed this issue have included a small patient population. A larger prospective studies exploring other toxicities besides colitis are needed and some of which are already underway (53).

Furthermore, recent studies show that variations in the gut microbiome have the potential to enhance the therapeutic response and reduce the irAEs associated with ICIs in multiple cancers (54, 55). The gut potential function of intestinal microbes as an immunemodulator (by increasing the anti-tumor effect and potentially reduce irAEs) is so considerable that some ongoing trials are investigating the possibility of combining them with anti PD-1/PD-L1 and anti-CTLA-4 drugs (21). The relationship of certain bacteria with vitamin B and poly-amine transport to the gastrointestinal tract may be the mechanism underlying the increased efficacy of immunotherapy in the background of the predominance of certain bacteria (56). The differences in the microbiome may apparently be responsible for toxicity or response, depending on the drug. Bacteroides fragilis, Burkholderia cepacia and the Faecalibacterium genus are associated with better response and lower incidence of colitis with anti-CTLA-4, while Bifidobacterium breve and longum, Akkermansia muciniphila, and Faecalibacterium prausnitzii are related with better outcomes with anti-PD-1/PD-L1 (56). The microbiome and its modifications may be responsible for the negative impact of some antibiotics on survival outcomes in patients receiving ICIs (57).

Another important biomarker that may be related to worse outcomes with anti-PD-1 treatment is sarcopenia. Several possible explanations exist, such as the implication of TGF-β and IL-6 and the development of chronic inflammation that results in cancer immune evasion through T cell exhaustion (58). Sarcopenia is related not only to poorer survival outcomes, but also to a higher incidence of irAEs (58). Besides, obesity has been linked with poorer outcomes with classic chemotherapy, but is apparently associated with improved outcomes in patients treated with ICIs (obesity paradox) (59). This association was especially marked when BMI and irAEs were considered in combination, meaning that the observed therapeutic benefit is further enhanced in the event of irAEs in the overweight population (60). Further studies are needed to analyse the cytokines that could be involved, as obesity is related to inflammation and metabolism, and its relationship with the hallmarks of cancer and immunotherapy requires investigation.

As mentioned above, the studies have that link potential biomarkers with irAEs are limited by their small sample size, and the unpredictable onset and frequency of these adverse events poses a challenge for the design of larger (much needed) prospective trials.

## Toxicity Itself as a Biomarker

Several studies have reported a positive association between the incidence of irAEs and the survival outcomes (6, 61, 62), while others have found no such association (63). A systematic review and meta-analysis has shown that grade ≥3 toxicities were correlated with a better overall response rate, but poor overall survival (64), while another has linked irAEs with better survival and response (7).

It is possible that certain immune-related adverse events possess a more direct relationship with anti-tumor efficacy than others (6), e.g., vitiligo in patients with melanoma. Thus, the irAEs could act as biomarkers themselves; however, since the intensity of irAEs cannot be modulated at present, nor can their severity be predicted before onset, irAEs cannot be uses as biomarkers of response. Doing so would jeopardize the patient by blindly exposing them to life-threatening adverse effects, owing to the lack of effective treatments that do not compromise the anti-tumor effect.

Furthermore, a few attempts were made at administering preventive treatment for the irAEs, which have been unsuccessful (65). However, it is debatable whether this could be attributed to the lack of efficacious preventive treatment or the utilization of a suboptimal biomarker.

## Discussion and Future Directions

This review assessed several potential biomarkers for severe toxicity evoked by ICIs; however, it could not conclusively identify a definite predictive biomarker for the timing of onset and occurrence of irAEs. Unfortunately, these data do not provide sufficient evidence to design a trial that can provide early treatment modalities for irAEs. Besides, considering the potential relationship between irAEs and tumor response, attempts to stop the onset of irAEs before they effect the modifications in the immune system needed to achieve longer survival may deprive patients of the potential long-term and ulterior benefits.

The future of oncological medicine lies in immunemodulation, and in line with this approach, other options should be explored for the treatment of irAEs that do not involve the use of corticosteroids, owing to their ambiguous effect on the anti-tumor activity of the immune system, if they are not administered at the optimal time, and substantial toxicity for patients (osteoporosis, infections, hypertension, hyperglycaemia, etc.) (66, 67).

Moreover, any discussion on the discovery of immunemodulators should include not only new combination drugs, but also physical activity (PA), vitamin D, and metabolism.

First, by virtue of reducing hypoxia and normalizing the tumor vasculature (68), PA can modify the TME and significantly reduce tumor aggressiveness (69). Moreover, PA induces transformations in the AKT and mTOR pathways, muscular IL-6, and mitochondrial function, which consequently inhibit tumor cell proliferation (68). Furthermore, PA stimulates NK cells by preparing the TME for their arrival, increasing the expression of NKG2D and NKp46 receptors (70). PA can increase the cytotoxic activity of T cells and macrophages, thus lowering the risk of metastasis (69). These modifications are also observed in patients who respond better to ICIs (71–73). Hence, it seems feasible that PA could act as a potential coadjuvant to immunotherapy, as already observed in pre-clinical models (74).

The potential of vitamin D as an immune modulator has also garnered interest. Vitamin D seem to benefit patients with autoimmune diseases; considering that irAEs share some characteristics with them, it is reasonable to assume that vitamin D may be useful for treating or even preventing their development (75, 76). Furthermore, vitamin D may play a role in the expression of PD-L1, owing to its vast immunemodulation potential. Moreover, as patients with cancer usually have vitamin D deficiency, regular testing, and examining its relationship with the development of irAEs could be an interesting direction for research. In fact, some ongoing studies have already focused on this aspect (ClinicalTrials.gov Identifier: NCT04615988).

Furthermore, the epigenetic role of metabolism on the immune system cannot be ignored (27). Exhausted T lymphocytes inhibit the AKT and mTOR pathways, stimulating fatty acid oxidation and increasing reactive oxygen species levels, and consequently, modifications in the exhausted T lymphocytes (77). However, active T lymphocytes mainly derive energy from glycolysis even in the absence of oxygen, which is inhibited by PD-L1, at least in chronic infections, but could also be relevant for neoplasms (77). The mitochondria play a fundamental role in this mechanism, and their potential involvement in the treatment for chronic infection and tumor control is being studied. Finally, the methylation pattern for exhausted T lymphocytes has been described, which seems to confer resistance to immunotherapy (78), making this mechanism a possible focus for future investigations.

In conclusion, it is clear that future research in the fields of immunotherapy and cancer is going to take a complex route, and an independent biomarker that can predict response, toxicity, or resistance to immunotherapy is not feasible. However, the results from studies on the new immune modulators may eliminate the need for high-dose corticosteroids. Their effects on the immunesystem, which are complex and sometimes contradictory, have an immenseimpact on toxicity, which cannot be allowed in this era of high precision medicine. We should guide our efforts to attempt to modulate the immune response to achieve better survival outcomes even without the development of irAEs.

## Author Contributions

AC-G wrote the manuscript and ML approved the final version. All authors contributed equally to the bibliographic research for this work. All authors contributed to the article and approved the submitted version.

## Funding

The research grant from the University Francisco de Vitoria to AC-G covered the expenses incurred for the publication of this article.

## Conflict of Interest

AC-G declares that despite the fact that the grant that covered the expenses of this article was provided by the University Francisco de Vitoria, the research course where she applied for the grant was financed by Takeda. ML was employed by Centro Estudios Biosanitarios.

## Publisher's Note

All claims expressed in this article are solely those of the authors and do not necessarily represent those of their affiliated organizations, or those of the publisher, the editors and the reviewers. Any product that may be evaluated in this article, or claim that may be made by its manufacturer, is not guaranteed or endorsed by the publisher.
